# Antibacterial, antioxidant and Immuno-modulatory properties in extracts of *Barleria lupulina* Lindl.

**DOI:** 10.1186/s12906-017-1989-4

**Published:** 2017-11-03

**Authors:** Reshma Kumari, Sumit Kumar, Ashwani Kumar, Kapil Kumar Goel, Ramesh Chandra Dubey

**Affiliations:** 1 Department of Botany & Microbiology, Gurukul Kangri University, Haridwar, 249404 India; 2 Department of Pharmaceutical Sciences, Gurukul Kangri University, Haridwar, 249404 India

**Keywords:** Immunomodulatory, Minimum inhibitory concentration, *Barleria lupulina*, Antioxidant activity, Phytochemical analysis

## Abstract

**Background:**

Antibacterial, immunomodulatory and antioxidant properties of aerial parts of *Barleria lupulina* Lindl were investigated in the present communication.

**Methods:**

The antibacterial, antioxidant and immunomodulatory properties of *B. lupulina* (methanol soluble leaf and stem extracts) was analyzed by minimum inhibitory concentration, total phenolic contents, DPPH radical scavenging activity, determination of toxicity, hemagglutination antibody titre, delayed type hypersensitivity and neutrophil adhesion test, respectively.

**Results:**

Methanol soluble leaf extract (MLE) contains more soluble bioactive compounds inhibiting the growth of five bacterial pathogens viz., *Staphylococcus aureus, Escherichia coli, Pseudomonas aeruginosa, Klebsiella pneumoniae,* and *Salmonella typhi* even at MICs of 1.25 and 2.5 mg/mL. Aqueous stem extract (ASE) was least effective while MLE was highly effective in inhibiting the growth and survival of bacterial pathogens. While testing the effect of these extracts in animal model, no mortality of albino rats was recorded by using MLE and MSE at the concentrations from 200 to 600 mg/kg of their body weight. The MLE showed significant result in agglutination reaction and induced paw edema volumes when compared with untreated group (control). Both MLE and MSE extracts significantly increased neutrophil adhesion with increase in doses of extracts. MLE was found to have more potent immune-stimulant properties than the MSE. High phenolic contents were found in MSE while lowest IC_50_ values were found in MLE in term of DPPH radical scavenging activity.

**Conclusions:**

Methanol soluble leaf and stem extract of *Barleria lupulina* contains antibacterial, antioxidants and immunomodulating phytochemical compounds that was effective for antibacterial, antioxidant and immunomodulatory properties. These may be used as synthetic drug.

## Background

Increase in the immune responsiveness is called immunostimulation and decrease in the immune responsiveness is called immunosupression. An immunomodulators may be defined as a substance which biologically can stimulate, suppress or modulate any of the components of the immune system or immune response. The essence of immunomodulation or phytochemical or biologically active compounds acting under various dose and time regimens displays an immunomodulating effects. Medicinal plants contains biologically active phytochemicals or bioactive agents that possess immune suppressing, immune stimulating, antimicrobial, antioxidating, anticancerous and other disease curative agents have their own standing and search for better agents exerting these activities if becoming the field of major interest all over the world. The use of traditional medicinal plants as remedy can be traced back to 600 BC in India. In addition natural biologically active phytochemical compounds have always been of great interest to innovative scientist working on infectious diseases or to improve immune system of human beings.

There are several medicinal plants are employed in different system of medicine throughout the world to improve the various immunological disorders in which *Barleria* (genus) is the one of the best that played important role in this concern. The genus *Barleria* is a large, polymorphic, widespread genus of herbs, shrubs and rarely climbers comprising approximately 300 species distributed worldwide. The greatest representation of this genus occurs in Africa and Asia, with its greatest centre of diversity in tropical East Africa.

The genus name *Barleria* is derived from the name of a Dominican monk and French botanist, Jacques Barrelier. Some species of *Barleria* are: *B. cristata, B. optusa, B. prionitis*, *B. acanthoides* *,* *B. aculeata* *,* *B. albostellata* *, Barleria greenii* ***,*** *B. repens* *,* *B. tetracantha* *,* *B. strigosa*, *B. lupulina,* etc. which are being used traditionally for a wide variety of ethnomedical properties. The name of *Barleria lupulina* varies from one region to another. The common names of *B. lupulina* are Hophead philippine violet, Bengali name is *Kanta Vishalyakarni*, Sanskrit name is *Neel Saireyak*, etc. It comprises of several medicinal properties including antibacterial and anti-inflammatory activities [15], anticancerous [33], anti-HSV-2 activities [35, 36], anti-clastogenic, anti-tumor [28, 29], antiarthritic [15], anti-diabetic, immunomodulatory [17]. Suksamrarn et al. [27] reported that several iridoid glucosides, bataine and alkaloids have also been reported from *B. lupulina* plant. Traditionally, leaves of this plant are used to treat snake bites, dog bites, swelling, bleeding wounds and rheumatism. The emergence of multi-drug resistance among bacterial pathogens throughout the world is kindled because of over prescription of antibiotics. The use of traditional medicinal plant is required as an alternative drug to cure various types of diseases.

No so much scientific data is available hence, in this research article attempt has been made to focus the experimental work on immunomodulation, antioxidation and antibacterial assay of methanolic and aqueous extracts of *Barleria lupulina.*

## Methods

### Collection of plant samples

Sample of *B. lupulina* leaves and stem were collected from the plant grown in Botanical Garden, Department of Botany and Microbiology, Gurukula Kangri University, Haridwar (India) in the month of September, 2014 and identified by the experts of the department (specimen identification No. Bot. & Micro/199/2016). The plant samples were washed with running tap water to remove the adhered dust, dirt and other foreign material and dried in shade at room temperature. The dried samples were separately homogenized to get fine powders which were stored in air tight container at room temperature for further studies.

### Preparation of crude extracts

The plant samples were powdered separately in shade and subjected to hot extraction in Soxhlet continuous extraction apparatus with methanol solvents for 48–72 h. The extracts were collected in a beaker, filtered separately and considered as the 100% concentrated stock extract. These extracts were evaporated by vacuum at reduced pressure. The extracts were then dried and weighed, and yield was calculated using the formula: yield % = X/Y × 100, where X = weight of the beaker with dried drug-weight of the beaker, and Y = total amount of the dried drug.

### Phytochemical analysis of plant extracts

#### Chemical tests

Methanolic leaf extract (MLE), stem extract (MSE) and aqueous leaf extract (ALE) of *B. lupulina* were subjected for qualitative analysis of phytoconstituents viz., alkaloids, proteins, aminoacids, flavonoides, carbohydrates, saponins, tannins, steroids, triterpenoids and cardiac glycosides following Trease and Evans [30].

#### Test for alkaloids (Dragendorff’s test)

Diluted 0.1 mL hydrochloric acid and 0.1 mL Dragendorff’s reagent were added in 2 mL of extracts separately in test tubes. After mixing, formation of orange brown coloured precipitate indicates the presence of alkaloids.

#### Test for steroids (Salkowski test)

Concentrated sulphuric acid (1 mL) was added separatetly to 10 mg of extracts and dissolved with 1 mL chloroform. A reddish brown colour exhibited by chloroform layer and green colour by the acid layer shows the presence of steroids.

#### Test for Saponins (foam formation test)

Extracts (1 mL) was separately diluted with distilled water up to 20 mL and vigorously shaken in a graduated cylinder for 15 min. Development of stable foam indicated the presence of saponins.

#### Test for cardiac glycosides (Raymond test)

Extracts (0.5 g) were separately dissolved in 50% ethanol. Then 1 mL of ethanolic m-dinitrobenzene solution and 2–3 drops of 20% NaOH solution were added separately and shaken properly. Formation of violet colour indicates the presence of glycosides.

#### Test for tannins (ferric chloride test)

Extract solutions (5 mL) were separately allowed to react with 1 mL of 5% ferric chloride solution. Appearance of greenish black colour indicates the presence of tannins.

#### Test for Flavonoids (lead acetate test)

Extracts (5 mL) were separatetly treated with 1 mL of 10% aqueous lead acetate solution. Formation of yellow coloured precipitate indicates the presence of tannins.

#### Test for proteins (Biuret test)

Extracts (3 mL) were treated with 1 mL of 10% sodium hydroxide solution and heated for 5 min. A drop of 0.7% copper sulphate solution was added to the mixtures. Appearance of violet colour indicates the presence of proteins.

### Determination of minimum inhibitory concentration (MIC) and survival of bacteria

MICs of both extracts were carried out to find out the lowest concentration of extract that inhibited the visible growth of test bacteria by standard tube dilution method [25].

#### In vitro antibacterial testing

The test bacteria, *Staphylococcus aureus, Escherichia coli, Pseudomonas aeruginosa, Klebsiella pneumoniae,* and *Salmonella typhi* were maintained on nutrient agar slants. Nutrient broth was used to test antibacterial activity of extracts. A loopful culture from the slants was separately inoculated in nutrient broth and incubated at 37 °C for 24 h. Fresh medium (20 mL) was seeded with 0.25 mL of 24 h old broth culture. The extract was dissolved in dimethyl sulphoxide (DMSO) to obtain 200 mg/mL stock solution. 0.2 mL solution of test material was added to 1.8 mL of the seeded broth to get and the first dilution i.e. 10^−1^ which was further serially diluted with 1 ml up to 8 tubes. A set of tubes containing only seeded broth was kept as a control and suitable solvent, DMSO controls were also maintained. Thereafter, the tubes were incubated at 37 °C for 24 h and results were recorded after incubation. The last tube with no visible growth of the bacteria was taken to represent the minimum inhibitory concentration (MIC) of the test sample and the result was expressed as mg/mL.

#### Bacterial survival

After completion of MIC test, 1 mL test sample was taken from each MIC tube (*E. coli, S. aureus, P. aeruginosa, S. typhi* or *K. pneumoniae*) and separated and poured into pre-sterile Petri plates containing nutrient agar media. Then the Petri plates were shaken and incubated at 37 °C for 48 h. After incubation as above bacterial colonies were counted as cfu/mL.

### Antioxidant activity

Both the extracts of *B. lupulina* were evaluated in vitro for antioxidant activity by using the following two methods:

#### Determination of total phenolic content

The total phenolic content present in both the extracts i.e. MLE and MSE were determined using a modified colorimetric Folin-Ciocalteu method [34]. This method is based on the principle of polyphenol that gives a blue colour chromogen in alkaline medium with Folin-Ciocalteu reagent at absorption maxima at 760 nm. Total phenolic content of the extract was determined using the Folin-Ciocalteu method [23]. Aliquots of 0.1 to 1.0 mL gallic acid solution were taken in test tubes and the volumes were adjusted to 9 ml with distilled water. Folin-Ciocalteu reagent (0.5 mL) was added to all tubes and allowed to stand for 30 min at room temperature. 0.5 mL of saturated sodium carbonate was then mixed and allowed to stand at 40 °C for 2 h. A linear graph of concentration Vs absorbance was prepared. Both extracts (2 mL each, 1 mg/mL) were treated similarly with 0.5 mL Folin-Ciocalteu reagent and allowed to stand for 30 min at room temperature. Then 0.5 mL saturated sodium carbonate (17%) was added in the reaction mixture, gently shaken and incubated at 40 °C for 2 h. Absorbance was measured at 760 nm with a spectrophotometer. Gallic acid (1 mg/mL) was used as standard reference; the total phenol content in the sample was expressed as mg of gallic acid equivalent to per gram of fresh extract.

### Determination of 1, 1-diphenyl-2-picrylhydrazyl (DPPH) radical scavenging activity

DPPH radical scavenging activity was assessed following Wong et al. [35] with slight modification. The method is based on the reduction of coloured solution of DPPH in presence of test sample. A free radical scavenging activity of both the extracts of *B. lupulina* was measured in terms of hydrogen-donating or radical scavenging ability using the stable radical DPPH. 100 mg test sample was dissolved in 50 mL of DMSO to get a concentration of 2 mg/mL. An equal amount of methanol was used as control (without extract). The solution was left in dark for 10–30 min. Then the absorbance of remaining DPPH was measured at 517 nm. Ascorbic acid was used as the reference compound. The radical scavenging activity was calculated by comparing the absorbance with the blank (100%) containing DPPH. Inhibitory Concentrations (IC_50_) values were determined for each extract using the following formula:

DPPH radical scavenging activity = (A_control_– A_sample_)/A_control_x 100.

Where, A_control_ is the absorbance of control reaction (without test sample), and A_sample_ is the absorbance in the presence of a plant extract.

### Determination of toxicity of plant extracts

Toxicity was determined by selecting different concentration of doses administered to albino rat (% mortality by using standard test). The new drugs have shown to possess the desirable pharmacological compounds during initial screening. It must undergo assessment of purity, potency, drug toxicity and other related properties leading to evolution of dosage for intensive evaluation of preclinical safety and bio-pharmaceutics testing before the start of early checkup of humans. The goal of the study was to select a dose which will achieve the highest exposure with the lowest dosage and yet to tolerate by the animal models involved for the period of toxicity studies. The research work on albino rats was carried out in department of pharmaceutical sciences, Gurukul Kangri Vishwavidyalaya, Haridwar after approval granted from the Institutional Animal Ethical Committee (Animal House Reg. No.: 1324/a/10/CPCSEA Govt. of India).

### Immuno-modulatory properties

The immune-modulatory properties of *B. lupulina* extracts were investigated to find out the new immune-modulators during stressful conditions of the animals. Immune-modulatory properties of leaf and stems were carried out by using standard animal model. For toxicity studies, three parameter viz., haemagglutination (HA) antibody titre, delayed type hypersensitivity (DTH), and neutrophil adhesion test were preformed. MLE and MSE extracts of *B. lupulina* were separately administered through oral route to four groups of albino rats. Each group had six albino rats with graded dosage (200 mg/kg, 400 mg/kg and 600 mg/kg body weight.) of extract. Mortality rate of rats were recorded after 7 days for treatment of HA antibody titres and DTH, and 14 days for neutrophil adhesion test [4].

#### Experimental animals

Six to 8 week-old healthy albino male rats (100–150 g) were kept in Animal House (Department of Pharmaceutical Science, Gurukula Kangri University) and maintained under standard environmental conditions, such as temperature (25 °C) and 12 h light and dark cycle. Six albino rats per cage were housed in a group in polypropylene cages (32 × 24 × 16 cm). They were fed standard pellet diet supplied by Hindustan Lever Ltd., Kolkata (India). Paddy husk was provided as bedding material by changing every day.

#### Dosage preparation

Each extract (1 g) of *B. lupulina* was suspended in 100 mL of 1% sodium carboxymethylcellulose (SCMC) to prepare the suitable dosages. All the extracts were administered orally by using metal canula. These suspensions were kept in refrigerator.

#### Antigen suspension (sheep red blood cells suspension)

Sheep red blood cells **(SRBCs)** was procured from Uttarakhand Sheep and Wool Development Board, Dehradun (India) in Alsever’s solution and washed twice with buffer saline (PBS). After the RBC count, the number of SRBCs was adjusted to a concentration of 0.5 × 10^9^ cells. RBCs were counted with the help of Neubauer’s counting chamber and RBC pipette. The RBC suspension was used to immunize and challenge the albino rats. The group 1 was fixed as a control group while the Group 2, 3 and 4 were used for the test substance i.e.*,* MLE and MSE extracts of *B. lupulina* in the 200, 400 and 600 mg/kg body weight, respectively. The duration of experiments was 7 days.

#### Hemagglutination antibody (HA) titres

The SRBCs agglutination assay was performed to study of the humoral antibody response against antigens. All the animals were immunized by injecting 0.5 × 10^9^ SRBCs, intra-peritoneal. The test samples of extracts were separately administered to all the rats continuously for 7 days. On the 7th day blood samples were collected from individual animal of the all groups by retro orbital bleeding into EDTA containing vials from serum was separated. Antibody level was evaluated by hemagglutination technique using 96 wells bottomed titre plate (12 × 8). The wells of titre plate were marked 1 to 12. In the first and last well, 25 μL of the serum collected from treated animals was added and left for inactivation at 56 °C for 30–40 min. Thereafter, 25 μL of PBS was added to all the wells except well number 12, and mixed properly. Then 25 μL of blood sample was taken from the first well, added to second well and serially diluted up to well number 10. Thereafter, 25 μL sample was taken out from well number 10 and discarded. Finally 25 μL of 1% suspension of SRBCs was added to all the wells and incubated at 37 °C for 1 h. Each well of the titre plate was examined for hemagglutination. The reciprocal of the highest dilution of the test serum giving agglutination was examined as the antibody titre. The data were analyzed statistically [13, 22].

#### Delayed type hypersensitivity (DTH)

On 7th days after oral administration of extract thickness of the right hind foot pad was measured with the help of Vernier caliper. Then animals were challenged by injecting 0.5 × 10^9^ SRBCs in right hind foot pad. Thickness of foot pad was measured at an interval of 24, 42 and 72 h. The differences between the pre-and-post challenges of foot pad thickness expressed were measured as the measure of delayed-type-hypersensitivity (DTH). The mean value obtained from treatment groups were compared with control group [13, 22].

#### Neutrophil adhesion test

On the 14th day of treatment, blood samples were collected (before challenge) by puncturing the retro-orbital plexus of the animals into heparinized vials and analyzed for total leukocyte counts (TLC) and differential leukocyte counts (DLC) by fixing blood smears and staining with field stain I and II Leishman’s stain [9, 32].

#### Total leukocyte count (TLC)

Blood sample was drawn and diluted with Turk’s fluid in WBC pipette, in which red cells were lysed without affecting the leukocyte population. Leukocytes were count by using an improved Neubauer’s counting chamber.

#### Differential leukocyte count (DLC)

Blood smear was prepared on a clean glass slide and different population of leukocytes was differentiated and identified based on the cell size, presence of granules, colour and shape of nucleus under the microscope using immersion oil. After initial counts, blood samples were inoculated with 80 mg/ml of nylon fibres for 15 min at 37 °C. The inoculated blood samples were again analyzed for TLC and DLC. The product of TLC and percentage of neutrophil provides by neutrophil index (NI) of blood sample. Neutrophil adhesion (%) was calculated by using the formula:

Neutrophil adhesion (%) = NI_u_ – Ni_t_/NI_u_ × 100.

Where, NI_u_ = neutrophil index of untreated blood sample; NI_t_ = neutrophil index of treated blood sample. The data were analyzed statistically.

## Results

### Preliminary Phytochemical analysis

Different phytochemicals were recorded in leaf and stem extract according to their solubility in different solvents. MLE consisted of alkaloids, saponins, tannins and flavonoids and proteins, whereas ALE consisted of saponins, flavonoids and sugars. But the MSE contained all phytochemicals except saponins, cardiac glycosides and amino acids (Table 1).

**Table 1:** Phytochemical constituents present in the extracts of different part of *B. lupulina*

Test	Extracts		
	MLE	ALE	MSE
Alkaloids	+	–	+
Steroids	–	–	+
Saponins	+	+	–
Cardiac glycosides	–	–	–
Tannins	+	–	+
Flavonoids	+	+	+
Proteins	+	–	+
Aminoacids	–	–	–
Sugars	–	+	+

‘+’ – Indicates the presence of phytochemical compounds

‘-’ - Indicates the absence of phytochemical compounds

### Minimum inhibitory concentration (MIC) of *B. lupulina* extracts and bacterial survival

MIC of MLE was 1.25 and 2.5 mg/mL against *E. coli, S. aureus, P. aeruginosa* and *S. typhi, K. pneumoniae,* respectively while MIC of ALE was 10 mg/mL at which growth of all the pathogens was inhibited except *S. aureus.* However, MIC of MSE was 2.5, 5.0 and 10.0 mg/mL against *P. aeruginosa*, *K. pneumoniae, E. coli, S. aureus* and *S. typhi,* respectively (Table 2).

**Table 2:** Minimum inhibitory concentration (MIC) extracts of *B. lupulina* and bacterial survival^a^

Extracts	MIC (mg/mL) of different bacterial species				
	*E. coli*	*S. aureus*	*P. aeruginosa*	*S. typhi*	*K. pneumoniae*
MLE	1.25 (1568)	1.25 (1632)	1.25 (1432)	2.5 (1579)	2.5 (1802)
ALE	10.0 (2496)	5.0 (2688)	10.0 (2400)	10.0 (2560)	10.0 (2640)
MSE	5.0 (1536)	5.0 (1760)	2.5 (1450)	10.0 (1655)	2.5 (1632)
ASE	10.0 (2478)	10.0 (2702)	10.0 (2409)	10.0 (2590)	10.0 (2726)
Control	(9248)	(9472)	(8960)	(9152)	(9248)

^a^Values in parentheses represent bacterial survival represented as total bacterial count (CFUs/mL)

The effect of methanolic and aqueous extracts ranged between 1.25 and 20.0 mg/mL. MLE resulted in the minimum number of colonies of *P. aeruginosa* (1432 cfu/mL) and the maximum number of *K. pneumoniae* (1802 cfu/mL) but ASE was least effective on growth of all pathogens as compared to both methanolic extracts. Maximum number of colonies of *K. pneumoniae* (2726 cfu/mL) and minimum of *P. aeruginosa* (2409 cfu/mL) were recorded after treatment with ASE. However, MSE was resulted in highest colonies against *S. aureus* (1760 cfu/mL) and lowest against *P. aeruginosa* (1450 cfu/mL) (Table 2).

### Antioxidant activity

#### Total phenolic content

The total phenolic contents in both methanolic extracts of *B. lupulina* are presented on the basis of percentage inhibition (Table 3). Phenolic contents present in extracts of different plant parts were compared with gallic acid as a standard because total phenolic contents of extracts is equivalent to phenolic content present in gallic acid. Phenolic contents in MLE and ASE of *B. lupulina* were recorded as 67.62 and 76.67 respectively as gallic acid equivalents. MSE extract consisted of more phenolic contents than the MLE (Table 3).

**Table 3:** Total phenolic contents (%) in different extracts of *B. lupulina*

Extracts	Extracts (%)
MLE	67.62
MSE	76.67
Gallic acid (Standard) = 73.56 μg/mL	
Blank reading = 0.763 nm	

#### DPPH (1–1-diphenyl 2-picryl hydrazyl) radical scavenging activity

The DPPH radical-scavenging activity (IC_50_) of MLE was 48.86 and that of MSE was 60.82. The results were compared with the IC_50_ value of ascorbic acid (25.75 μg/mL). IC_50_ value is the effective concentration at which antioxidant activity is 50%. On the basis of IC_50_ value it may be explained that MLE consisted of better free radical scavenging activity than the MSE extract of *B. lupulina*. Antioxidant activity of different extracts were observed in the order MLE > MSE. A higher DPPH radical scavenging activity was recorded at lower IC_50_ value (Table 4).

**Table 4:** Effect of leaf extracts of *B. lupulina* in DPPH antioxidant model

Concentrations (μg/mL)	Percentage of inhibition of leaf extracts	
	MLE	MSE
20	12.3198	3.408
30	19.031	15.99
40	33.340	34.469
50	59.131	58.06
IC_50_ value	48.86	60.82

Blank reading = 0.763 nm

### Toxicity of extracts

In the present investigation no mortality of albino rats was recorded in both treatments of extracts (Table 5).

**Table 5:** Toxicity analysis of methanolic leaf and stem extracts of *B. lupulina* on albino rats

Animal group^a^	Extract dose (mg/kg b. wt)/Mortality (%)	
	MLE	MSE
I	200/0	200/0
II	400/0	400/0
III	600/0	600/0
Control group^b^	10/0	10/0

^a^Each group contain 6 albino rats

^b^Vehicle control = Pure saline

### Immuno-modulatory properties

In this study MLE and MSE of *B. lupulina* were analysed for their immune-modulatory properties based on HA titres, DTH response, and neutrophil adhesion methods.

#### Hemagglutination antibody (HA) titre

HA titre-based study showed that MLE and MSE of *B. lupulina* significantly (*p* > 0.01) improved humoral response to challenge of SRBC_S_. HA titres were tested after 7 days of oral administration of different concentrations of both the extracts. The mean values of HA titres of both extracts were compared with vehicle control (Table 6). Albino rats fed with MLE of *B. lupulina* for 7 days at different doses (200, 400 and 600 mg/kg body weight) showed agglutination of HA titre as 23.21, 35.0 and 44.0 cells, respectively as compared to control group of rats (21.52). A significant increase in HA titre was observed with dosages of 200, 400 and 600 mg/kg body weight. MSE at different doses (200, 400 and 600 mg/kg body wt.) showed 24.50, 28.0 and 36.0 cells, HA agglutination titre respectively. Administration of MSE showed a significant agglutination titre in comparison to control. Both extracts showed dose-dependent increase in HA titre when compared to control albino rats. The MLE gave the better result than MSE (Table 6).

**Table 6:** Effect of methanolic leaf and stem extracts of *B. lupulina* on hemagglutination antibody (HA) titre

Animal group^a^	Extract Dose(mg/kg body wt)	HA titre (mean ± SD)	
		MLE	MSE
Control	10 mL/kg Control^b^	21.52 ± 0.57	
I	200	23.21 ± 1.26	24.50 ± 1.20
II	400	35.0 ± 2.91	28.0 ± 2.91
III	600	44.0 ± 3.02	36.0 ± 3.12

Values are mean ± S.D., *p* < 0.01 significant

^a^Each group contain 6 albino rats

^b^Vehicle control = Pure saline

#### Delayed type hypersensitivity (DTH) response

DTH response to SRBCs was calculated as a measure of paw oedema thickness (in mm) at the concentrations of 200, 400 and 600 mg/kg body weight of each animal and compared with vehicle control group which was orally injected with 10 mL/kg body weight of normal saline for 7 days. An increase in paw oedema thickness of each group of animal was recorded after 24, 48 and 72 h of treatment. Albino rats treated with MLE showed a significant (*p* > 0.01) difference with control group. After 24 h, the test values for all doses (200, 400 and 600 mg/kg body weight) were 0.28, 0.30 and 0.36 mm, respectively, whereas after 48 h, the values were 0.26, 0.30 and 0.35 mm, respectively. However, after 72 h, the thickness of paw oedemas of rats was 0.25, 0.29 and 0.34 mm, respectively (Table 7).

**Table 7:** Effect of methanolic leaf and stem extracts of *B. lupulina* on delayed type hypersensitivity response

Animal group^a^	Extract Dose(mg/kg b.wt)	DTH response (mm) (Mean ± SD) after 24, 48 and 72 h treatment					
		MLE			MSE		
		24 h	48 h	72 h	24 h	48 h	72 h
Control	10 ml/kg Control^b^	0.20 ± 0.10	0.20 ± 0.01	0.18 ± 0.01	0.20 ± 0.10	0.20 ± 0.01	0.18 ± 0.01
I	200	0.28 ± 0.01	0.26 ± 0.03	0.25 ± 0.01	0.22 ± 0.05	0.23 ± 0.01	0.20 ± 0.05
II	400	0.30 ± 0.02	0.30 ± 0.03	0.29 ± 0.01	0.24 ± 0.01	0.23 ± 0.03	0.22 ± 0.08
III	600	0.36 ± 0.05	0.35 ± 0.57	0.34 ± 0.57	0.32 ± 0.05	0.31 ± 0.02	0.30 ± 0.08

Values are mean ± S.D., *P* < 0.01 significant

^a^Each group contain 6 albino rats

^b^Vehicle control = Pure saline

After 24 h, the treated albino rat groups (200, 400 and 600 mg/kg body weight) showed the increased values (0.22, 0.24 and 0.32 mm) of paw thickness in the MSE than in control (0.20 mm). However, less or equal values were recorded after 48 and 72 h of treated with different concentrations of MSE. These values increased after 48 and 72 h when compared with control group of rats (Table 7). All the rats groups treated with MLE and MSE showed more significant (*p* > 0.01) results than the control group of rats. All extracts resulted in dose-dependent increase in the paw thickness. The values of paw oedema thickness gradually decreased with the increase in time. The DTH response in MSE was found more significant than the MLE. Apart from humoral response, there was a significant (*p* > 0.01) increase in DTH response or cell-mediated immunity. Both extracts of *B. lupulina* showed an increase in delayed type hypersensitivity in all the treated group of rats when compared to vehicle control group of rats (Table 7).

#### Neutrophil adhesion (%) test

Neutrophil adhesion test was performed by the adhesion of neutrophils to nylon fibres and results were compared with that of control group of rats. MLE with different doses (200,400 and 600 mg/kg body weight) showed neutrophil adhesion by 14.72, 16.84 and 21.97%, respectively, whereas it was 7.32% in control. Nylon fibre treated blood samples of different extracts showed lesser number of total leukocyte cells than untreated blood samples (Table 8). MSE at different doses viz., 200, 400 and 600 mg/kg body weight showed neutrophil adhesion by 13.5, 16.25 and 19.84%, respectively, whereas it was 7.32% in case of vehicle control group of rats (Table 9).

**Table 8:** Effect of methanolic leaf extract of *B. lupulina* on neutrophil adhesion

Animal group^a^	Extract Dose(mg/kg body wt.)	TLC (10^3^ cells/ μL) [A]		Neutrophil (%) [B]		Neutrophil Index [A × B]		Neutrophil Adhesion (%)
		UB	FTB	UB	FTB	UB	FTB	
Control	10 ml/kgControl^b^	4.50 ± 2.01	4.35 ± 8.40	7.52 ± 2.10	7.21 ± 3.05	33.84 ± 10.01	31.36 ± 5.21	7.32 ± 1.91
I	200	5.00 ± 3.20	4.85 ± 2.10	6.80 ± 5.12	6.00 ± 0.97	31.0 ± 0.52	29.10 ± 0.89	14.72 ± 2.56
II	400	6.98 ± 1.25	6.95 ± 0.13	7.40 ± 2.50	7.10 ± 3.12	51.65 ± 1.60	42.95 ± 0.23	16.84 ± 3.02
III	600	8.81 ± 0.12	7.20 ± 0.57	8.40 ± 0.57	8.02 ± 0.91	74.00 ± 1.24	57.74 ± 1.60	21.97 ± 1.18

Values are mean ± S.D., p < 0.01 singnificant

^a^Each group contain 6 albino rats

^b^Vehicle control = Pure saline

*UB* Untreated blood

*FTB* Fiber treated blood

**Table 9:** Effect of methanolic stem extract of *B. lupulina* on neutrophil adhesion

Animal group^a^	Extract Dose(mg/kg body wt.)	TLC (10^3^ cells/ μL) [A]		Neutrophil (%) [B]		Neutrophil Index [A × B]		Neutrophil Adhesion (%)
		UB	FTB	UB	FTB	UB	FTB	
Control	10 ml/kgControl^b^	4.50 ± 2.01	4.35 ± 8.40	7.52 ± 2.10	7.21 ± 3.05	33.84 ± 10.01	31.36 ± 5.21	7.32 ± 1.91
I	200	6.52 ± 0.29	6.23 ± 0.33	8.12 ± 1.98	7.03 ± 1.50	52.98 ± 4.13	43.79 ± 1.23	13.5 ± 5.48
II	400	7.21 ± 0.33	7.12 ± 0.72	10.52 ± 2.00	8.92 ± 2.52	75.84 ± 3.09	43.79 ± 1.23	16.25 ± 4.27
III	600	8.55 ± 0.17	8.08 ± 0.15	11.20 ± 4.50	9.50 ± 5.12	95.76 ± 4.96	76.76 ± 5.26	19.84 ± 5.20

Values are mean ± S.D., p < 0.01 significant

^a^Each group contain 6 albino rats

^b^Vehicle control = Pure saline

*UB* Untreated blood

*FTB* Fiber treated blood

## Discussion

### Phytochemical analysis

Different types of phytochemicals constituents are found in different medicinal plants viz., alkaloids, saponins, tannins, flavonoids, steroids, glycosides, etc. However, the chemical constituents of plants may differ in different environmental conditions or stress conditions. Extractions of phytochemical compounds also depend on the solubility of organic solvents. In the present investigations, MLE of *B. lupulina* consisted of more phytochemicals than the MSE. Similarly, the MLE contained more phytoconstituents than the ALE (Table 1).

Doss et al. [6] have also reported the presence of alkaloids, tannins, steroids, and flavonoids only in petroleum ether, chloroform and methanol soluble extracts. Mazumder et al. [16] have reported the presence of flavonoid, sterol, alkaloid and phenolic compounds in methanol extract of *B. lupulina*. Similarly, the aqueous fraction of *B. lupulina* contains alkaloids, reducing sugars and phenolic compounds. But our results are better than those reported by them. Phytochemical analysis of ethanolic extract of *B. lupulina* revealed the presence of steroid, glycoside, terpenoid, flavonoid, tannin and carbohydrate as reported by Sur and Das [29] and Mazumder et al. [17]. Presence of more or less similar phytoconstituents in varying amount in different solvents has also been reported by the other workers [6, 16, 20, 21]. These results are similar to that of ethanolic extract recently reported by Kumari and Dubey [11, 12].

#### Antibacterial properties

The MLE was more inhibitory to growth of all bacterial pathogens than the other extracts. Differential inhibitory properties of plant extracts would have been possible due to accumulation of bioactive compounds more in leaves than the other part of the plant. Further, the presence of more chemicals in methanol-soluble extract than the other extracts may be explained to be due to their solubility more in methanol than the water extracts.

Similarly antibacterial activity by disc diffusion method of methanol soluble extract of *B. lupulina* with MIC of 0.125 mg/mL against *E. coli* and *S. aureus* has been reported by Doss et al. [6]. But our results on antibacterial properties are better that reported by Doss et al. [6]. It may be possible due to adoption of different approaches for chemical extraction and antibacterial testing against enteric pathogens. Ethanolic extract at 2.5 mg/mL MIC inhibited the growth of *E. coli, S. aureus* and *P. aeruginosa* as compared to that of aqueous extracts. All four pathogens were inhibited at 10 mg/mL except *S. aureus* (5.0 mg/mL) using water extracts [12]. However Doss et al. [6] did not reported MIC values against test pathogens.

Bacterial survival at a specific MIC of *B. lupulina* has not earlier been reported so far. But we report the bacterial survival in different MIC tubes. The bacterial counts decreased with treatment of leaf and stem extracts. After completion of experiment, less counts of *P. aeruginosa* was recorded showing its maximum sensitivity to extracts. In contrast, *K. pneumoniae* showed the maximum number of CFUs which indicates it to be resistant to extracts tested.

#### Antioxidant activity

The role of free oxygen radicals in many ailments and their prevention by using antioxidants are receiving much attention in recent years. Each medicinal plant contains different phenolic contents and every compound possesses different amount of antioxidant activity. Phenolic acid is a major class of phenolic compounds, which occur widely in the plant kingdom, especially in fruits and vegetables. Mostly phenolics possessing antioxidant activity are mainly phenolic acids and flavonoids. The results obtained from present studies demonstrate that *B. lupulina* may be a good source of bioactive substances with multifaceted activity. The MSE of *B. lupulina* showed the highest amount of antioxidant activity but BLE displayed less antioxidant activity. Further MSE had higher phenolic contents than gallic acid (standard).

A similar relationship between phenolic and antioxidant activity has also been reported in ethyl acetate fraction of *B. lupulina* (EFBL) showing the highest value of TPC, followed by methanol soluble extract, and acetone soluble fraction phenolic and flavonoid content, which are responsible for antioxidant property of plants [16]. We found better results than Mazumder et al. [16] and report the antioxidant properties even in least amount of extracts. The phenolic compounds may contribute directly to antioxidative action [7].

All extracts of *B. lupulina* showed significant scavenging effects with increasing concentration from 20 to 50 μg/mL due to the DPPH radicals. Decrease in absorption at 517 nm shows the reduction ability of DPPH. Positive DPPH test suggests that the presence of free radical scavengers in plant samples. Moreover, the radical scavenging activity of extracts could be related to the nature of phenolic compounds, thus contributing to their electron transfer/hydrogen donating ability [14]. A higher DPPH radical scavenging activity is associated with a lower IC_50_ value. However, synthetic antioxidants, like ascorbic acid was found to be more potent for providing the hydrogen electron transfer ability than the samples of the plant extract. Similar work has been reported earlier by Mazumder et al. [16] who found varying IC_50_ values of different organic solvent soluble extracts. They adopted different extraction procedures due to which IC_50_ values also differed from our findings. We found 48.86 μg/mL IC_50_ value in MLE that was the lowest value of DPPH radical, and the highest from hydrogen peroxide assay [16].

#### Immuno-modulatory properties

Antibodies are produced by B lymphocytes which are central to humoral immune responses. Among immunomodulation isolates, IgG and IgM are the major immunoglobulins which are associated with complement activation, opsonization, neutralization of toxins, etc. [18]. The augmentation of the humoral immune response to SRBCs by plant extracts as evidenced by an increase in the antibody titre in albino rats indicated the enhanced responsiveness of T and B lymphocyte involved in antibody synthesis [2]. The high values of HA titre obtained after MLE feeding indicated the achievement of humoral immunity through immune-stimulation. Animals treated with different doses of methanol extract of *Curculigo orchioides* have also shown an increase in the haemagglutination titre in a dose-dependent manner [1].

Antibodies function as effectors of the humoral response by binding to antigens and facilitating its elimination by cross-linking to form clusters are more readily ingested by phagocytic cells. An increased agglutination titre to SRBCs (antigen) in our study indicates the enhanced responsiveness of macrophages and T-cell and B-cells involved in antibody synthesis which have also been reported by Benacerraf (1978).

The MLE expressed significantly (*p* > 0.01) the agglutination titre in a better way than the MSE. This indicates that these extracts suppress humoral immune response and interfere with antibody formation affecting the agglutination titre against SRBC. In the present study, MLE enhanced the level of IgM and IgG, and influenced B-cells which in turn synthesized the increased antibody molecules linked with SRBC leading to subsequent agglutination. Similar type of work has been reported by Damre et al. [5]. They found that the flavonoid fraction of *Tephrosia purpurea* (FFTP) affected the cellular and humoral functions and macrophage phagocytosis in mice. It also produced a significant, dose-related decrease in sheep erythrocyte-specific haemagglutination antibody titre [5]. Similar findings with aqueous and alcoholic extracts of *Echinacea purpurea* [8], alcoholic extract of *Isatis cappadocica* and aqueous extract of *Clausena excavate* have been reported with increased HA titre of IgM and DTH responses.

Cell-mediated immunity is a part of the process of graft rejection, tumor immunity, and immunity to many intracellular infections of microorganisms causing chronic ailments. Delayed type hypersensitivity **(**DTH) requires the specific recognition of given antigen by activated T-lymphocytes, which subsequently proliferate and release cytokines. The MLE of *B. lupulina* significantly increased the DTH response. The MLE and MSE also influenced the T-cell activity significantly, which in turn increase the vascular permeability, induced vasodilatation, macrophage accumulation and activation. This finally results in increase of concentration of lytic enzymes for more effective killing, which is ultimately reduced the paw odema after 48 and 72 h of treatment to the albino rats. Mazumder et al. [12] also discussed a similar result on the effect of MEBL (300 mg/kg body weight) on spleen weight, WBC count, spleen leukocyte count and percentage increase in paw volume on delayed type hypersensitivity consisting footpad thickness. Methanolic extract of *B. lupulina* (600 mg/kg body weight) showed the positive effect (*P* < 0.01) on all parameters than the dose of 300 mg/kg body weight. The increase in blood leukocytes count, weight of spleen and splenic leucocytes count indicate an uplift of immune status. A significant inhibition in SRBCs-induced delayed-type hypersensitivity reactions has also been achieved through oral feeding of FFTP (10–40 mg/kg) [5].

Suba et al. [26] reported the anti-inflammatory efficacy of methanol extract of aerial parts of *B. lupulina* in acute and subacute inflammation models on albino rats. The methanolic extract exhibited significant inhibition of carrageenin and serotonin induced paw oedema volumes when compared with the untreated (control) group. A similar result was also found in our investigation in case of paw oedema volumes that *B. lupulina* bears the ability to increase DTH response.

In vivo inflammatory models have been examined using carrageenan-induced paw oedema and ethyl phenylpropiolate-induced ear oedema models in rats. A powerful dose-dependent inhibitory effects induced by *B. lupulina* and *C. nutans* extracts has been reported [31]. In the present study, the efficacy of neutrophil function was examined by immunizing the rats as an animal model. Neutrophil adherence was analysed by the initial total leukocyte and differential leukocyte count (DLC) from the blood samples of rats. Results indicate that TLC and neutrophil count (%) in fibre treated blood sample is lower than the untreated blood samples. Circulations of immune cells are essential for maintaining an effective immune defense network. The TLC was found to be increased in extract immunized group of albino rats, which may be due to the fall in the corticosterone levels and increased glucocorticoides levels, which affect the circulation of pattern of immune cells [24]. Margination of neutrophils from the blood stream required a firm adhesion, which is mediated through the interactions of β_2_ integrins (cytokines) present on the neutrophils [19].

Adherence to nylon fibres was increased in the methanol soluble leaf and stem extracts of *B. lupulina* immunized group because of the regulation of β_2_ integrins and also by decreased cortisterone level. Reports suggest that oral administration of methanolic extracts of *B. lupulina* significantly increase the neutrophil adherence to nylon fibres. Similar work has been reported by Benschop et al. [3] who found that an increased glucocorticoids level may also lead to this decrease in the neutrophil adherence. Neutrophil constituents create phagocytosis, an essential arm of the host to defence against foreign antigens.

Methanol-soluble leaf and stem extracts showed significant amount of neutrophil adhesion, which was different in each of other extracts. This may be due to the concentration of bioactive phytochemical present in specific extract. Both extracts of *B. lupulina* (200–600 mg/kg, per oral) evoked a significant result in neutrophil adherence. Similar work has also been reported by Ghule and yeole [10] on immunomodulatory activities of the iridoids fraction (i.e. n-butanol fraction of methanol extract) from *Barleria prionitis* aerial parts (IFBp) (100 and 200 mg/kg p.o.) that evoked a significant increase in neutrophils and neutrophils adhesion to nylon fibres. Oral administration of IFBp augmented the humoral immune response to SRBCs, as evidenced by increase in antibody titres and dose potentiated the delayed-type hypersensitivity reaction induced by SRBCs in mice.

## Conclusion

In the present investigation methanol soluble leaf and stem extracts of *B. lupulina* were found a potent immunostimulant, stimulating both the specific and non-specific immune mechanisms. It is also contains antibacterial as well as antioxidant properties due to phytochemical compounds. This suggests that these extracts bearing antibacterial, antioxidant and immuno-modulatory properties act as an effective candidate which may be used for the prevention of different ailments like autoimmune diseases.

## Abbreviations

ALE: Aqueous Leaf Extract
ASE: Aqueous Stem Extract
DLC: Differential Leukocyte Counts
DMSO: Dimethyl Sulphoxide
DPPH: 1, 1-diphenyl-2-picrylhydrazyl
DTH: Delayed Type Hypersensitivity
HA: Hemagglutination Antibody
MIC: Minimum Inhibitory Concentration
MLE: Methanolic Leaf Extract
MSE: Methanolic Stem Extract
NI: Neutrophil Index
SCMC: Sodium Carboxymethylcellulose
SRBC: Sheep Red Blood Cells
TLC: Total Leukocyte Counts
